# Association between Parental Workaholism and Body Mass Index of Offspring: A Prospective Study among Japanese Dual Workers

**DOI:** 10.3389/fpubh.2016.00041

**Published:** 2016-03-17

**Authors:** Takeo Fujiwara, Akihito Shimazu, Masahito Tokita, Kyoko Shimada, Masaya Takahashi, Izumi Watai, Noboru Iwata, Norito Kawakami

**Affiliations:** ^1^Department of Social Medicine, National Research Institute for Child Health and Development, Tokyo, Japan; ^2^Department of Mental Health, The University of Tokyo Graduate School of Medicine, Tokyo, Japan; ^3^National Institute of Occupational Safety and Health, Kanagawa, Japan; ^4^Department of Nursing, Nagoya University, Aichi, Japan; ^5^Department of Psychology, Hiroshima International University, Hiroshima, Japan

**Keywords:** child obesity, child overweight, parenting, working environment, work–life balance

## Abstract

The purpose of the study was to investigate the association between parental workaholism and child body mass index (BMI) among Japanese dual-income families. In 2011, 379 dual-income families from urban Tokyo with children aged 0–5 years were recruited for a baseline survey, and 160 (42.2%) were followed up in 2012. Demographics, workaholism, work demands, work control, time spent with children, and parental and child weights and heights were assessed using a questionnaire. Structural equation modeling was performed to determine the association between maternal and paternal workaholism in 2011 and child BMI in 2012, considering the mediating effects of time spent with children. Paternal workaholism showed a direct significant positive association with child BMI after 1 year (standardized coefficient: 0.19; *p* < 0.001), while maternal workaholism was not associated with child BMI. Both maternal and paternal time spent with children did not mediate the association. Paternal work demands showed a strong positive association with workaholism but paternal work control did not. Paternal, but not maternal, workaholism was associated with an increase in child BMI over 1 year. Interventions that target workaholism by reducing paternal work demands might be effective in preventing overweight in offspring.

## Introduction

Over the past three decades, childhood obesity has increased worldwide, including Japan (1, 2). Children who are obese or overweight are more likely to be obese or overweight in adulthood (3). Further, childhood obesity can lead to a wide range of diseases, including hypertension (4), dyslipidemia (5), type 2 diabetes mellitus (6), sleep apnea (7), renal dysfunction (8), and depression (9). As dietary and exercise habits are difficult to change in adulthood, early interventions for childhood obesity are important to prevent obesity later in life (1, 10).

Determinants of childhood obesity vary from genetic to non-genetic factors (10). Non-genetic factors include bottle feeding (11), a sedentary lifestyle and longer periods of watching television (12, 13), and poor eating habits, such as larger intakes of sugar-sweetened beverages (14) or fast food (15). Recent studies suggest that these non-genetic factors related to childhood obesity might be determined by family factors, such as maternal employment or longer maternal working hours in conjunction with a rise in dual-income families (16–20). Moreover, both maternal and paternal working styles, such as the type of work schedule, have been investigated as determinants of child obesity (21–23).

However, these previous studies did not reveal which component of parental working style is associated with child obesity. We hypothesized that parental workaholism, which is defined as “the tendency to work excessively hard and to be obsessed with work” (24), can explain the association between parental working style and child body mass index (BMI). Higher work demands could induce higher parental workaholism, which might reduce the length of time spent with children and/or reduce the quality of that time (19). Given that, previous studies have reported that parenting or parent–child interactions had effects on child BMI (25–27), and time spent with children, which is a proxy of parent–­­child interaction, could also be associated with child BMI. Few studies have empirically revealed the association between parental workaholism and child BMI that take into account the impact of work demands and control on the time spent with children.

Further, it is necessary to investigate this hypothesis among dual-income families because time spent by the mother and time spent by the father interact with each other (28). The impact of parental workaholism and the mediating effects of time spent with children on child obesity can be investigated better among dual-working families. Among child-rearing families in Japan, the percentage of dual-working families is gradually increasing, from 29.7% in 1985 to 44.3% in 2013 (29). Here, we aim to investigate the association between parental workaholism and child BMI among dual-working families in Tokyo, Japan, using a prospective cohort study design.

## Materials and Methods

### Sample

This study is a part of the Tokyo Work–Family Interface Study (TWIN) II, a large cohort study that commenced in 2011. We invited participants of TWIN I (30), the study we initially started, to participate in the TWIN II survey (*N* = 321 families). Additionally, we also approached all day care centers (*N* = 22) in another ward, Meguro city, with the permission of Meguro’s day care division. We sent our research plan and consent forms to the principals of all day care centers. All principals agreed to participate and sent the research plan and consent forms to parents. After checking the signed consent forms from those parents who agreed to participate, we directly sent out questionnaires to participating parents’ addresses (*N* = 357 families). Therefore, questionnaires were distributed to all parents (*N* = 678 families) in 2011, and 413 families responded to the questionnaire (response rate: 60.9%). Further, follow-up questionnaires were distributed in 2012, and 229 responses were received (follow-up rate: 55.4%). Of these, valid responses on workaholism and both parental and child BMI were received from 125 families (54.6%).

### Measures

Workaholism was assessed in the baseline questionnaire during the fiscal year (FY) of 2011. The questionnaire was based on the Japanese version of the Dutch Workaholism Scale, which showed good reliability and validity (31). The scale consists of two subscales: working excessively and working compulsively. Both subscales comprised five items with a 4-point Likert scale (1 = “totally disagree” and 4 = “totally agree”).

In addition, work demands and work control were assessed using the Brief Job Stress Questionnaire (32). Both scales were composed of three items (e.g., for work demand, “I am asked to do an excessive amount of work” and for work control, “I have influence over the pace of my work”), with a 4-point Likert scale (1 = “disagree” and 4 = “agree”). The sum of all item responses was used for analysis.

Time spent with children was assessed using the following question: “How long do you spend with your child per week?”; responses included units of hours per week. The response was used as a continuous variable. Parental and child weights and heights were assessed using a self-reported questionnaire (i.e., self-reported for parental weight and height and parent-reported for child weight and height). Previous studies among Japanese have proven the high accuracy of self-reported child weights and heights (33, 34), as well as that for adults (35). BMI (kilogram/square meter) was calculated as weight (kilogram) over height squared (square meter). Child BMI was adjusted for age and sex, and BMI *z*-scores were calculated based on World Health Organization standards (36). Parental BMI was collected from the baseline questionnaire in FY 2011, and child BMI was collected both at baseline in FY 2011 and at follow-up in FY 2012. Other covariates, including parental characteristics, such as age, annual household income, occupation, and status of maternity or paternity leave, were collected for both mothers and fathers, and child’s sex and age were also assessed at baseline in FY 2011.

### Analysis

First, to confirm whether workaholism and work demands and control reflected parental occupation, the mean scores of each work-related scale were compared between occupations of mothers and fathers using analysis of variance. Second, structural equation modeling was used to determine the association between maternal and paternal workaholism in 2011 and child BMI in 2012, considering the mediating effects of parental BMI, time spent with children, and child BMI in 2011. Further, we incorporated the association of both work demands and control related to workaholism with time spent with children in the model. Stata SE 13 (StataCorp, College Station, TX, USA) was used for the analysis.

## Results

Table 1 shows the characteristics of the sample. Mean paternal and maternal ages were 39.6 (SD: 6.3) and 38.0 (SD: 4.5) years, respectively. Mean BMI for fathers was 23.2 (SD: 3.0) and 20.1 (SD: 2.1) for mothers, which is consistent with the average BMI for this age group in Japan (37). Annual household income was skewed to the right, i.e., the sample was relatively wealthy, with more than half reporting an annual income of more than 10 million yen (equivalent to USD 80,000 as of 2015), as reported by fathers. Interestingly, mothers reported a lower annual household income than fathers. The type of paternal occupation was distributed almost equally for non-manual (52.8%) and manual (40.8%) jobs, and only one participant was taking paternity leave at the time of the survey. For mothers, manual occupations were in the majority (55.2%) and non-manual occupations accounted for 36.8%, while 7.2% of participants were taking maternity leave at the time of the survey. Regarding children’s characteristics, boys were dominant at 61.6%, and child ages ranged between 2 and 95 months. Mean *z*-scores of child BMI for both FY 2011 and 2012 were 0.002 and −0.095, respectively, suggesting that participating children were not skewed to either overweight or underweight.

**Table 1:** **Characteristics of the sample (*N* = 125)**.

				Mean or *n*	SD or %	Min.	Max.
Parents	Age (years)	Father		39.6	6.3	28	62
		Mother		38.0	4.5	27	47
	BMI (kg/m^2^)	Father		23.2	3.0	18.6	33.9
		Mother		20.1	2.1	15.4	27.5
	Time spent with the child (hours/week)	Father		31.1	21.9	0	100
		Mother		53.4	25	8.5	140
	Annual household income (million yen)	Paternal response	<5	8	6.4		
			5–<10	50	40		
			10–<15	38	30.4		
			15+	25	20		
			No answer	4	3.2		
		Maternal response	<5	20	16.0		
			5–<10	48	38.4		
			10–<15	35	28.0		
			15+	18	14.4		
			No answer	4	3.2		
	Occupation	Father	Non-manual	66	52.8		
			Manual	51	40.8		
			Paternity leave	1	0.8		
			Other	7	5.6		
		Mother	Non-manual	46	36.8		
			Manual	69	55.2		
			Maternity leave	9	7.2		
			Other	1	0.8		
Child	Sex	Male		77	61.6		
		Female		48	38.4		
	Age (months)			44.4	21.4	2	95
	*z*-score of child BMI	FY 2011		0.002	1.05	−2.39	2.37
		FY 2012		−0.095	1.19	−4.21	2.46

Associations between occupation and workaholism, work demands, and work control for both fathers and mothers are shown in Table 2. Among fathers, occupation did not significantly differentiate the scores for workaholism, work demands, and work control (all *p* > 0.2). However, among mothers, occupation differentiated the scores for workaholism and work demands (both *p* < 0.05). More specifically, mothers on maternity leave showed a higher score of workaholism and work demands than mothers currently working in non-manual or manual occupations (both *p* < 0.05). Further, mothers with manual occupations had lower scores for work control (*p* < 0.05).

**Table 2:** **Work-related psychometric scales by occupation (*N* = 125)**.

		Total	Occupation				
			
			Non-manual (*n* = 66 for father, *n* = 46 for mother)	Manual (*n* = 51 for father, *n* = 69 for mother)	Paternity/maternity leave (*n* = 1 for father and *n* = 9 for mother)	Others (*n* = 7 for father and *n* = 1 for mother)	*p*
				
		Mean (SD)	Mean (SD)				
Workaholism	Father	21.8 (6.1)	22.6 (6.1)	21.5 (5.9)	21.0 (NA)	18.0 (6.9)	0.27
	Mother	20.9 (5.6)	20.4 (5.5)	20.8 (5.3)	24.9 (6.5)	10.0 (NA)	0.03
Work demands	Father	8.8 (2.0)	9.1 (2.0)	8.5 (2.1)	8.0 (NA)	8.4 (1.6)	0.46
	Mother	8.4 (2.0)	8.0 (2.0)	8.5 (1.9)	10.1 (1.4)	3.0 (NA)	0.0014
Work control	Father	9.1 (1.9)	9.3 (1.7)	8.7 (2.1)	9.0 (NA)	9.7 (2.0)	0.36
	Mother	8.24 (2.0)	8.7 (1.8)	7.8 (2.0)	8.9 (2.4)	11.0 (NA)	0.058

**Figure 1:**
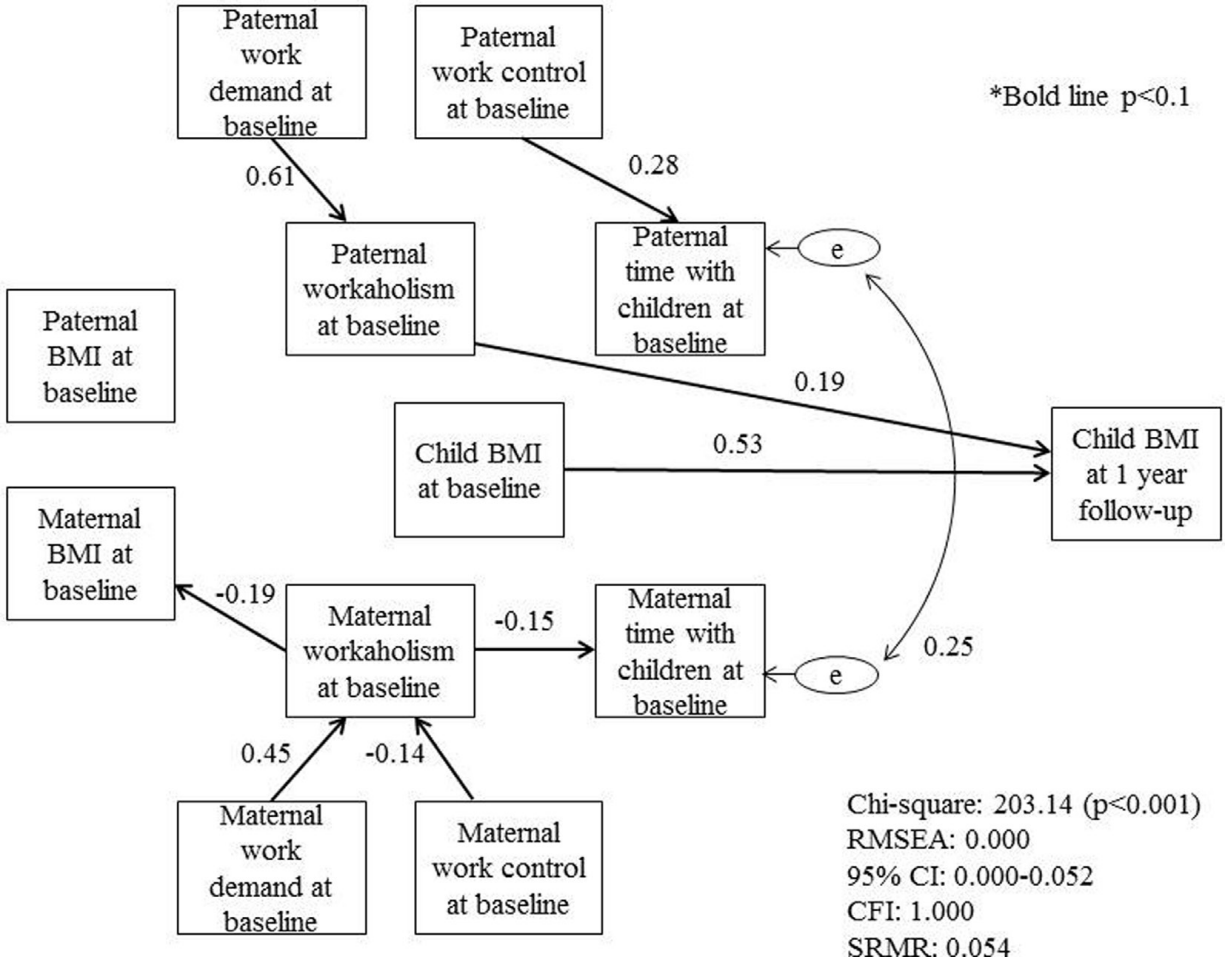
**Hypothesized model with standardized coefficients (*N* = 125)**. Dotted line represents non-significant paths (*p* > 0.1). Chi-square = 203.14 (*p* < 0.001), root mean square error of approximation (RMSEA) = 0.000, 95% confidence interval (CI) 0.000–0.052, and comparative fit index (CFI) = 1.000.

Figure 1 shows the structural equation model that estimates the causal path of workaholism and *z*-scores of child BMI 1 year after baseline. The hypothesized model showed acceptable goodness-of-fit indices: chi-square = 203.14 (*p* < 0.001), root mean square error of approximation (RMSEA) = 0.000, 95% confidence interval = 0.000–0.052, and comparative fit index (CFI) = 1.000. As shown, paternal workaholism was directly associated with *z*-scores of child BMI 1 year after baseline (standard coefficient: 0.19, *p* < 0.05), that is, time spent with children or paternal BMI did not mediate the association. Paternal work demands were positively significantly associated with paternal workaholism (standard coefficient: 0.61, *p* < 0.05) while paternal work control was associated with time spent with children (standard coefficient: 0.28, *p* < 0.05), as expected. Interestingly, maternal workaholism was not associated with *z*-scores of child BMI 1 year after baseline, neither directly nor indirectly. That is, although maternal workaholism was inversely associated with maternal BMI (standard coefficient: −0.19, *p* = 0.061) and time spent with children (standard coefficient: −0.14, *p* = 0.074), these were not associated with *z*-scores of child BMI. Maternal work demands and control were both associated with maternal workaholism (standard coefficient: 0.45 and −0.14, *p* < 0.05 and *p* = 0.078, respectively). Further, time spent with children showed strong correlations for both mothers and fathers (standard coefficient: 0.25, *p* < 0.05).

## Discussion

To the best of our knowledge, this is the first study to report that paternal workaholism, but not maternal workaholism, was directly associated with an increase in child BMI over 1 year, regardless of paternal or maternal time spent with children or parental BMI. Our findings suggest that paternal workaholism, which was positively associated with work demands, was linked with childhood overweight or obesity 1 year after baseline.

Previous studies reported that maternal working hours were associated with childhood obesity (16–20), and few studies investigated the impact of the paternal work environment. Champion et al. revealed that non-standard paternal work schedules, defined as “shift work and working after 1800 hours (including work at home), overnight, or on weekends,” showed a higher odds ratio for child overweight or obesity than did a standard working schedule after adjustment for maternal working schedules among families with 9-year-old children in Australia (21). This is consistent with the findings of the current study because workaholism is associated with working at home or work-to-family conflict (38), which was included as a non-standard work schedule, such as night-time shift working. Further, Morrissey reported that both maternal and paternal employment intensities are associated with higher child BMI during preschool (22). As our study focused only on dual-income families, the impact of paternal workaholism might be found only if both the mother and father are working. Nonetheless, because of the rise of women returning to work after having children, the findings suggest the importance of paternal workaholism on child BMI, which is usually not considered in the prevention of childhood obesity.

The possible mechanisms of how paternal workaholism directly increases child BMI require further research. As time spent with children did not mediate the association, the quality rather than the quantity of time spent might be more important. For example, fathers with higher scores for workaholism might be less likely to spend time engaging with their children in energetic activities, such as tag or sports, because of a high work-to-family conflict (38). Alternatively, paternal workaholism might be associated with a specific personality, which has a tendency toward a permissive or disengaged parenting style, as this parenting style leads to child overweight and obesity (25). In addition, family interaction during mealtimes, which has an association with child obesity (26), might be associated with workaholism. Further study is needed to investigate the association between paternal workaholism and the quality of time spent with children, parenting style, or paternal interaction with children during mealtimes to elucidate the relationship between paternal workaholism and child BMI.

The lack of association between maternal workaholism and child BMI might be surprising, considering the previous studies that reported maternal working hours were associated with childhood obesity (16–20). Possibly, maternal working may not be associated with the poor quality of parenting, such as providing unhealthy food that might induce obesity of children (39), because of social pressure embedded in Japanese culture. Generally, mothers are expected to be the main caretaker of her child regardless of her working status. This trend is reflected by the high rate of maternity leave (83.4%) and low rate of paternity leave (2.8%) in 2010 (40). As the current study is the first to report on the null association between maternal workaholism and child BMI, further replication of the study is needed to confirm the association.

This study has several limitations. First, the sample size of this study was small; more studies with larger sample sizes are needed to validate our findings. Second, although we tried to capture a population-based sample, the participants may not be a representative sample, given the relatively low response and follow-up rate. Third, as parents reported their child’s weight and height, measurement error might exist, even if parental reports of child height or weight have been shown to be accurate (33, 34).

Based on our findings, we suggest engaging employers of fathers for reducing work demands and increasing work control through workplace education. As shown in Figure 1, work demands were associated with workaholism. Reducing paternal work demands might be necessary to reduce paternal workaholism, which in turn could decrease child BMI if the mother is also working. On the contrary, although paternal work control was associated with paternal time spent with children, increasing work control to prevent child obesity may not be effective because time spent with children was not associated with child BMI. In a systematic review of prevention programs for childhood obesity, study settings were limited to school, home, primary care facilities, or the community, and no study was implemented in a workplace setting (41). Further studies in the workplace are needed to address this gap to prevent childhood obesity.

## Conclusion

Paternal, but not maternal, workaholism was associated with an increase in child BMI after 1 year. Interventions that address paternal workaholism by reducing paternal work demands might be effective to prevent overweight in children. Further study is needed to replicate these findings using a larger sample and to elucidate the mechanisms underlying the association between paternal workaholism and child BMI.

## Ethical Approval

All procedures performed in studies, involving human participants, were in accordance with the ethical standards of the Institutional Research Committee of the University of Tokyo and with the 1964 Declaration of Helsinki and its later amendments or comparable ethical standards.

## Author Contributions

TF and AS conceived the study; AS, MTo, and KS collected data; TF analyzed and wrote the first draft; and AS, MTo, KS, MTa, IW, NI, and NK finalized the manuscript. All authors approved the final version of the manuscript.

## Conflict of Interest Statement

The authors declare that the research was conducted in the absence of any commercial or financial relationships that could be construed as a potential conflict of interest.
